# Gentamicin-Attenuated *Leishmania infantum* Vaccine: Protection of Dogs against Canine Visceral Leishmaniosis in Endemic Area of Southeast of Iran

**DOI:** 10.1371/journal.pntd.0002757

**Published:** 2014-04-17

**Authors:** Hamid Daneshvar, Mohammad Javad Namazi, Hossein Kamiabi, Richard Burchmore, Sarah Cleaveland, Stephen Phillips

**Affiliations:** 1 Research Center of Tropical and Infectious Diseases, Kerman University of Medical Sciences, Kerman, Iran; 2 Sabzevar Medical University, Sabzevar, Iran; 3 Parasitology Department, Kerman University of Medical Sciences, Kerman, Iran; 4 Institute of Infection, Immunity and Inflammation, College of Medical, Veterinary and Life Sciences, University of Glasgow, Glasgow, United Kingdom; 5 Boyd Orr Centre for Population and Ecosystem Health, Institute for Biodiversity, Animal Health and Comparative Medicine, University of Glasgow, Glasgow, United Kingdom; 6 School of Life Sciences, College of Medical, Veterinary and Life Sciences, University of Glasgow, Glasgow, United Kingdom; University of Iowa, United States of America

## Abstract

An attenuated line of *Leishmania infantum* (*L. infantum* H-line) has been established by culturing promastigotes *in vitro* under gentamicin pressure. A vaccine trial was conducted using 103 naive dogs from a leishmaniosis non-endemic area (55 vaccinated and 48 unvaccinated) brought into an endemic area of southeast Iran. No local and/or general indications of disease were observed in the vaccinated dogs immediately after vaccination. The efficacy of the vaccine was evaluated after 24 months (4 sandfly transmission seasons) by serological, parasitological analyses and clinical examination. In western blot analysis of antibodies to *L. infantum* antigens, sera from 10 out of 31 (32.2%) unvaccinated dogs, but none of the sera from vaccinated dogs which were seropositive at >100, recognized the 21 kDa antigen of *L. infantum* wild-type (WT). Nine out of 31 (29%) unvaccinated dogs, but none of vaccinated dogs, were positive for the presence of *Leishmania* DNA. One out of 46 (2.2%) vaccinated dogs and 9 out of 31 (29%) unvaccinated dogs developed clinical signs of disease. These results suggest that gentamicin-attenuated *L. infantum* induced a significant and strong protective effect against canine visceral leishmaniosis in the endemic area.

## Author Summary

A 24 month vaccine trial was conducted using 103 leishmania free dogs in an area of southeast Iran endemic for visceral leishmaniosis. The dogs were vaccinated with gentamicin-attenuated line of *Leishmania infantum*. No local and/or general indications of disease were observed in the vaccinated dogs immediately after vaccination with an attenuated line of *Leishmania infantum*. Nine out of 31 (29%) unvaccinated dogs, but none of those vaccinated, were positive for presence of *Leishmania* DNA by PCR. In western blots, sera from 10 out of 31 (32.2%) unvaccinated dogs, but none of the sera from vaccinated dogs, recognized the 21 kDa antigen of *Leishmania infantum* wild-type. One out of 46 (2.2%) vaccinated dogs and 9 out of 31 (29%) unvaccinated dogs developed clinical signs of disease. The attenuated *Leishmania infantum* induced a significant and strong protective effect against *Leishmania infantum* infection in the field.

## Introduction

*Leishmania infantum* (*L. infantum*) is a causative agent of visceral leishmaniasis (VL), which is a severe and frequently lethal protozoan disease of humans and dogs. Canine visceral leishmaniosis (CVL) is widely distributed in large areas of Europe, South America, the Middle-East, Central Asia, China, and Africa, particularly in the countries of the Mediterranean Basin [1], [2]. In Iran, at least seven endemic foci in dogs have been identified including the Baft district in the southeast of the country where there is a high seroprevalence in domestic dogs [3]. Dogs are the principal reservoir of *L. infantum* and can be an important threat to public health. Control of the disease in dogs has been shown to reduce the human incidence [4], [5]. Although there have been a number of vaccine trials, there is currently no effective and completely safe vaccine against any form of leishmaniasis. A successful vaccine against *Leishmania* is most likely to be either an attenuated line or a subunit vaccine based on antigens with demonstrable protective function [6], [7]. Subunit and attenuated vaccines can be highly effective and induces protection against pathogen [8], [9], [10]. We previously reported that a cultured attenuated line of *L. infantum*, identified as *L. infantum* H-line, was selected by culturing promastigotes *in vitro* under pressure of gentamicin [11]. Gentamicin, which has frequently been added to cultures of *Leishmania* to prevent bacterial contamination [12], [13], is an aminoglycoside that interacts with RNA in prokaryotic cells [14]. The precise mechanism of bactericidal activity of aminoglycosides is not fully understood, but some hypotheses include disruption of ribosomal activity by breaking up polysomes, misreading of mRNA during protein synthesis and production of abnormal or nonfunctional proteins. Comparative proteomics profiling of the attenuated line identified key changes in parasite thiol-redox metabolism [15]. Thiol-redox metabolism is crucial for *Leishmania* which is exposed to an oxidative burst when they encounter their mammalian macrophage host cell [16]. *L. infantum* H-line is more susceptible to oxidative stress, and thus a change in thiol-redox metabolism in this line may explain its loss of virulence [15]. *L. infantum* H-line invaded but was unable to survive within bone marrow derived macrophages of BALB/c mice *in vitro* [11]. Moreover, the attenuated line failed to spread to, and within, the visceral organs of BALB/c mice and dogs over a 12 week observation period [17]. Immunohistochemical investigation showed no parasites in the popliteal lymph node (PLN) of immunized dogs whereas there were parasites in the PLN of 60% of dogs infected with *L. infantum* WT [18]. No clinical signs and histopathological abnormalities were found in the dogs immunized with the attenuated line of parasite over 2 years post-immunization [17], [19]. Dogs immunized with the attenuated line parasites elicited a Th1 response and were protected against experimental CVL [19]. We previously reported that Western blot analysis of antibodies to the 21 kDa antigens of *L. infantum* H-line and WT might be a useful technique for distinguishing between dogs vaccinated with *L. infantum* H-line and dogs naturally infected with *L. infantum* WT in epidemiologic studies [20].

In the present study, for the first time, we show the impact of *L. infantum* H-line vaccine against natural infection in dogs in a highly endemic area of Iran over a 24 month follow-up.

## Materials and Methods

### Parasites

Promastigotes of *L. infantum* JPCM5 (MCAN/ES/98/LIM-877), were cultivated in complete haemoflagellate minimal essential medium (HOMEM) (GIBCO) supplemented with 10% (vol/vol) heat-inactivated fetal calf serum (HI-FCS) (Labtech International). *L. infantum* H-line was generated in the same medium supplemented with 10% (vol/vol) HI-FCS and gentamicin at 20 µg/ml (Sigma) [11]. Stationary phase promastigotes of the attenuated line were harvested after 48 subpassages and a suspension at a concentration of 5×10^8^ cells/ml in PBS was prepared.

### Study site

**Figure 1:**
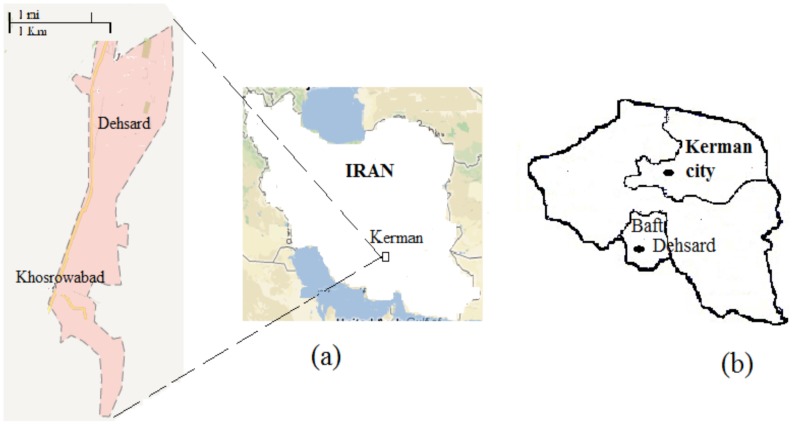
Maps representing (a) the geographical locations where this study was carried out, in 3 villages of Baft County, Kerman Province, in the southeast of Iran. (b) Kerman city 225 Km of northwest of Dehsard, the leishmaniosis non-endemic area. Healthy German shepherd cross dogs obtained from Kerman city and brought into Dehsard, the leishmaniosis endemic areas.

The field study was conducted in 3 villages, Dehsard, Khosrowabad and Dehsarar of Baft County (56.2147°E, 28.2727°N), Kerman Province, in the southeast of Iran (Fig. 1a). The area has a desert climate and the total annual rainfall is 309 mm with a minimum of 3 mm in July and maximum of 120.9 mm in April. The minimum mean monthly relative humidity is 26% (June) and the maximum is 56% (January). Initially, 77 household dogs were examined for clinical signs of the disease and tested for the presence of specific anti-*Leishmania* antibody by an immunofluorescence assay (IFA).

### Ethics statement

A vaccine trial was conducted on 103 dogs (55 vaccinated and 48 unvaccinated). The protocol for vaccination of the dogs had been reviewed and approved by the Medical Ethics and Animal and Use Care Committee of the Kerman Medical University (study protocol number KA/89/15), in accordance with the Guide for the Care and Use of Laboratory Animals Eighth Edition. The animals were kept under typical local conditions of food and housing and sampled with the owners' consent. All dogs with clinical signs of disease were sacrificed to avoid unnecessary suffering.

### Study design and animal

On the basis of the rate of seropositivity detected in the 77 dogs tested (see above), and to anticipate a number of dogs being lost during the follow-up period of 24 months, a total 103 dogs were used in this study. At follow-up prior of starting, we expected 60% seropositive in the unvaccinated group and 20% seropositive in vaccinated group, with a confidence level of 0.95 and power of 0.9, and ratio of 1.29 sample size (i.e. n2/n1 equivalent to 45/35). This estimated a sample of 31 in unvaccinated group and 40 in vaccinated group. Ten unvaccinated dogs and 13 vaccinated dogs were considered to lose at follow-up prior in this study. One hundred and three healthy male German shepherd cross dogs from non-endemic areas (Kerman city 225 Km northwest of Dehsard) (Fig. 1b) between 6–18 months old were used. All of the animals were negative for presence of leishmanial DNA and serum specific anti-*Leishmania* IgG antibody. The dogs had previously been vaccinated against canine parvovirus and rabies and were also treated with the anthelmintic drugs praziquantel and pyrantel. The weight and age were recorded for each dog and they were randomly divided into 2 groups (55 dogs in vaccinated group and 48 dogs in unvaccinated group). The dogs in the vaccinated group were injected subcutaneously (s.c) with 100 µl of the suspension of stationary phase promastigotes in the foreleg of the animals. The unvaccinated dogs were injected subcutaneously with 100 µl of PBS also in the foreleg. The dogs were transferred into the endemic area over a period of 1.5 months before June 2010 and re-homed at households within the endemic area. Information about the risk of the procedures was given to persons who became owners of dogs. We included vaccinated and control dogs in each house whenever possible, in order to match their degree of exposure to natural infection.

### Follow-up

The dogs were followed up over 24 months, from June 2010 to cover four sandfly seasons, which occur in June and September in the endemic areas of the southeast of Iran [21]. The efficacy of the vaccine was evaluated by clinical examination and serological and parasitological analyses. Active disease surveillance measures were implemented in each of the study villages. A trained worker was located in the Health House in the village, and together with our team had responsibility for disease monitoring. The follow-up was performed at 3, 6, 9, 12, 18, 20 and 24 months after starting trial. The peripheral blood samples were taken for complete blood cells (CBC) count and biochemical parameters including serum total protein, serum albumin and serum globulin. The clinical signs of disease were classified according to a modified version of leishvet guidelines as described previously [22]. Briefly, Stage I, Mild disease, animals exhibiting peripheral lymphadenomegaly or papular dermatitis, creatinine <1.4 mg/dl, non-proteinuric, negative to low levels of antibody. Stage II, Moderate disease, animals, which apart from the signs listed in stage I, may exhibit diffuse or symmetrical cutaneous alterations (exfoliative dermatitis/onychogryphosis, ulcerations (planum nasale, footpads, bony prominences, mucocutaneous junctions), anorexia, weight loss, fever, and epistaxis, mild non-regenerative anemia, hyperglobulinemia, hypoalbuminemia, normal renal profile, creatinine <1.4 mg/dl, low to high levels of antibody. Stage III, Severe disease, animals which apart from the signs listed in stages I and II, may exhibit signs originating from immune-complex lesions such as vasculitis arthritis and glomerulonephritis, chronic kidney disease, creatinine 1.4–2 mg/dl, medium to high levels of antibody. Stage IV, Very severe disease, animals which, apart from the signs listed in stages I, II and III, may exhibit signs pulmonary thromboembolism, or nephrotic syndrome and end stage renal disease (creatinine >5 mg/dl) with medium to high levels of antibody. Twenty six out of 103 (25.2%) dogs left or died from a disease unrelated to leishmaniosis over the 24 month period follow-up. All unvaccinated dogs were sacrificed by intravenous injection of thiopental sodium 33% (5 ml/kg) [23] at the end of the study. Five ml of peripheral blood were taken from the foreleg vein of each dog in EDTA for isolating parasite DNA for PCR test, and 2 ml for separation of sera for IFA and Western blotting test. The samples were stored at −20°C.

### Detection specific anti-*Leishmania* total IgG, IgG1, and IgG2 antibodies

Specific anti-*Leishmania* total IgG antibody was measured by IFA as described previously [24]. Briefly, slides were coated with washed promastigotes of *L. infantum* WT and air dried. Slides were incubated with twofold dilutions of the test samples in humid moist conditions for 60 min at 37°C. Excess antibody was washed off the slides and bound antibody was detected using fluorescein isothiocyanate (FIT) conjugated sheep anti-dog IgG (Sigma), diluted at 1∶400 in PBS-0.01% Evans blue. Specific anti-*Leishmania* IgG1 and IgG2a antibodies were measured by ELISA as described previously [19]. Briefly, serum was prepared from the clotted blood of dogs at 20-month post follow-up and stored at −20°C. Each well of flat-bottom microtitre plates was coated with 1 µg of soluble *Leishmania* antigen in 0.1 M carbonate buffer pH 9.6 and incubated at 4°C overnight. Following 3 washes in PBS (pH 7.4) containing 0.05% Tween-20, the plates were blocked with 200 ml of blocking buffer (1% BSA in PBS), and incubated at 37°C for 1 h. After 3 washes, 100 µl of serially diluted serum sample (1∶100 starting dilution in PBS/BSA 1%) was added to the wells and incubated for 2 h at 37°C. Bound antibodies were detected by 50 µl/well goat anti-dog IgG1 conjugated to HRP at 1∶500 dilution and for detection of IgG2, 50 µl/well sheep anti-dog IgG2 conjugated to HRP at 1∶5000 dilution (Bethyl Laboratories, Montgomery, TX, USA). The plates were incubated at 37°C for 1 h and subsequently washed 6 times. One-hundred µl of TMB substrate were added to each well. The reaction was stopped after 15 min incubation at room temperature using H_2_SO_4_ (0.5 M) (50 µl). Absorbances were measured at 405 nm on an ELISA reader.

### Western blotting

The Western blot technique was applied as described previously [25]. Briefly, stationary phase promastigotes of *L infantum* H-line or wild-type parasite (1×10^7^ cells per lane) were washed with ice-cold PBS three times, and disrupted by sonication. An equal volume of sample buffer [0.1 M Tris (Merck), 12% sodium dodecyl sulfate (Merck), 10% glycerol (Merck), 5% β-mercaptoethanol (Merck), 0.1% bromophenol blue, pH 8.0] was mixed and the solution denatured at 95°C for 5 min. Promastigote lysates were fractioned individually on a 12% SDS-PAGE gel and subsequently transferred onto a nitrocellulose membrane (Sigma-Aldrich). The blots were individually incubated with 1∶50 diluted sera in PBS containing 3% skimmed milk at room temperature for 18 h. The blots were incubated with 1∶10000 diluted goat anti-dog IgG-heavy and light chains antibody horseradish peroxidase (HRP) conjugated (Bethyl Lab. Inc) in PBS containing 5% skimmed milk at room for 2 h. The blots were washed as above, incubated with ECL Plus chemiluminescent substrate (GE Healthcare), and exposed to X-ray film.

### DNA extraction and diagnostic PCR

The possible presence of leishmanial DNA was assayed in peripheral blood and PLN necropsy. DNA was extracted (Promega, Columbus, OH, USA), according to the manufacturer's instructions and stored at −20°C until use. PCR amplification was carried out in 50 µl reaction volumes using 0.5 pmol of the kinetoplastid-specific primers K13A (5′-GTGGGGGAGGGGCGTTCT-3′) and K13B (5′-ATTTTACACCAACCCCCAGTT-3′) [26]. The amplification products were analysed by 1.5% agarose gel and visualized under UV light. A positive control containing genomic DNA of *Leishmania*-infected dog and negative control without template DNA were included.

### Report of VL cases from vaccine trial areas

VL cases were monitored from the records of each of the pediatric wards of Afzelipour Medical Centre at Kerman University of Medical Sciences. As VL cases might occasionally be referred to other hospitals, further information on VL cases were obtained from Centre for Disease Control and Prevention in Kerman Medical University.

### Statistical analysis

Statistical analyses were performed with statistical package EpiTool (available at http://epitools.ausvet.com.au) to determine the number of animals in the vaccine trial. For quantitative data, Student's t-test was used to calculate differences the levels of IgG between two groups. Comparison between the levels of IgG1 and IgG2 antibodies was performed using Paired sample t-test. Chi-square test was used to examine the relationship between the number of dogs which IFA titers of IgG antibody were at >1∶100 between 2 groups. Fisher's exact test was used to calculate difference leishmanial DNA between 2 groups. Data are expressed as the mean ± standard deviation mean (SDM) for each group. Differences were considered significant when *P*<0.05.

## Results

### Screening of household dogs before vaccine trial

Seventy seven household dogs, living in the endemic areas, were examined for clinical signs of the disease and tested for presence of specific anti-*Leishmania* IgG antibody. Specific anti-*Leishmania* antibody by IFA was found in 31 out of 77 (40.2%) of the householder dogs (≥1∶100). Sixty two out of 77 (80.5%) animals were asymptomatic.

### Follow-up

The efficacy of the vaccine was evaluated after 4 sandfly transmission cycles by clinical examination, and serological and parasitological analyses. A vaccine trial was conducted on 103 dogs (55 vaccinated and 48 unvaccinated). No local indications including swelling, and pain at the injection site and no general indications of disease including anorexia, apathy, vomiting and diarrhoea were observed after the vaccine administration. Twenty three dogs (9 vaccinated and 14 unvaccinated) (22.3%) left the study after a change in residence or disappearance. Three unvaccinated dogs (2.9), died. Two of these dogs had to be put down because of accidental injury and one died from a disease unrelated to leishmaniosis.

### Specific anti-*Leishmania* total IgG, IgG1, and IgG2 antibodies

**Figure 2:**
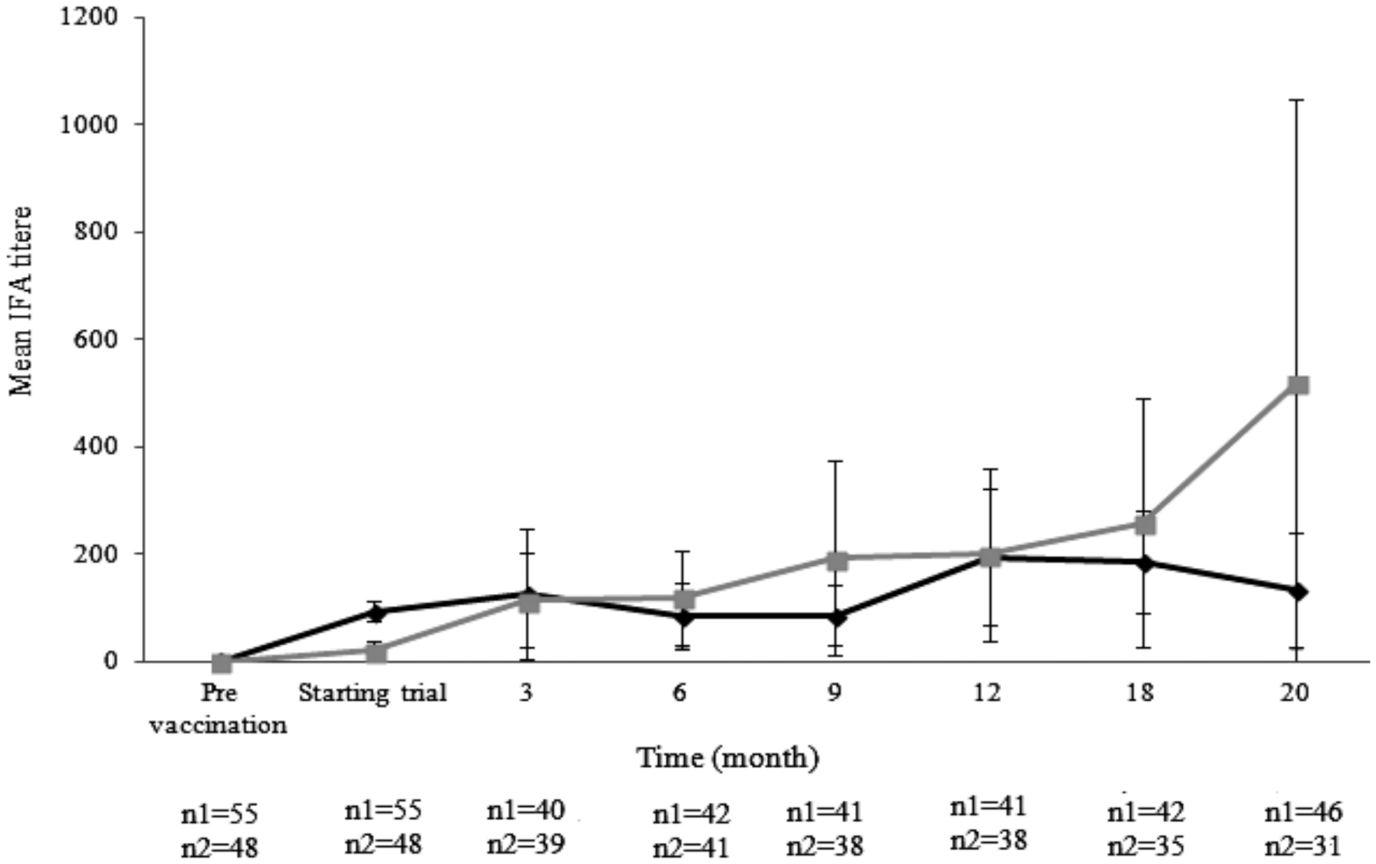
Mean levels of specific anti-*Leishmania* total IgG antibody in the sera of vaccinated (♦) and unvaccinated dogs (▪). The dogs in the vaccinated group were injected with the *L. infantum* H-line and then transferred into the endemic area over a period of 1.5 months. Antibodies were detected by IFA pre-vaccination and over a 20 month period follow-up. The number of vaccinated dogs (n1) and unvaccinated dogs (n2) were assayed at each individual time point. After 1.5 months transferred into the endemic area. Each point represents average ± SDM.

All vaccinated dogs gave positive titers of specific anti-*Leishmania* antibodies whereas, all but 2 unvaccinated dogs were seronegative over the 3 month follow-up (Fig. 2). Fluctuations of the mean levels of antibody were observed in the sera of vaccinated dogs over the 20 month follow-up (Fig. 2) but did not rise. In contrast, the mean levels of antibody increased in the sera of unvaccinated dogs over the same period (Fig. 2). There was a significant difference between mean levels of antibody in the sera of vaccinated and unvaccinated dogs (*P*<0.001). The cut-off for which animals were considered seropositive was established to be a positive IFA results at serum dilutions of >1∶100. As shown in Table 1, twelve out of 31 (38.7%) unvaccinated dogs and 2 out of 46 (4.3%) vaccinated dogs were seropositive at >1∶100. The rest of dogs, 19 out of 31 (61.2%) unvaccinated dogs and 44 out of 46 (95.7%) vaccinated dogs were seropositive ≤1.100 over the 24 month follow-up. The number of unvaccinated dogs which were seropositive at >1∶100 was significantly higher than vaccinated dogs (*P*<0.0005). Specific anti-*Leishmania* IgG1 and IgG2 antibodies were present in the sera of dogs vaccinated with *L. infantum* H-line, predominantly of the IgG2 subclass. In the sera of vaccinated dogs, the level of IgG1 was significant lower than the level of IgG2 (*P*<0.001) whereas, the level of IgG1 was significantly higher than the level of IgG2 in the sera of unvaccinated dogs (*P*<0.05).

**Table 1:** Clinical stages of disease, IFA titers of specific anti-*Leishmania* antibody, presence of leishmanial DNA in LN and western blot analysis were evaluated in all dogs from both groups.

Group	Animal code	Clinical stage	IFA titer	PCR	Recognize 21 kDa antigen of *L. infantum* WT
Vaccinated	V20	*	1∶400	−	−
dogs	V33	-	1∶400	−	−
**Total**		**0/46 (0%)**	**2/46 (4.3%)**	**0/46 (0%)**	**0/46 (0%)**
Control	C2	-	1∶400	−	−
dogs	C5	II	1∶800	+	+
	C9	III	**	+	+
	C12	-	1∶400	−	+
	C16	-	***	+	−
	C27	II	1∶800	+	+
	C28	I	1∶400	−	+
	C46	II	1∶1600	+	+
	C48	II	1∶1600	+	+
	C49	II	1∶1600	+	+
	C52	II	1∶1600	+	+
	C59	II	1∶1600	+	+
	C61	-	1∶400	−	−
**Total**		**9/31(29%)**	**12/31(38.7%)**	**9/31(29%)**	**10/31(32.2%)**

*: Moderate weight loss unrelated to leishmaniasis.

**: Dog was seropositive at 1∶3200 and sacrificed at 19-month post follow-up.

***: Dog was seropositive at 1∶100.

### Western blot analysis

**Figure 3:**
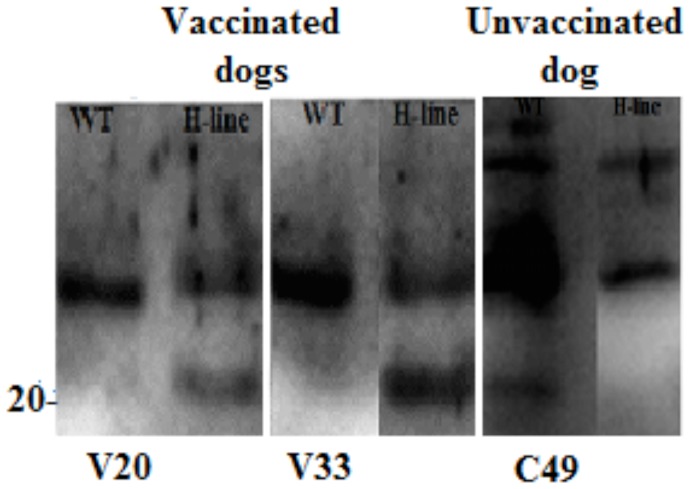
Western blot analysis of antibodies to *L. infantum* antigens vaccinated dogs, V20 and V33, and unvaccinated dogs infected naturally with *L. infantum* WT, C49. Sera of vaccinated dogs recognized the 21*L. infantum* H-line (H-line), but not of *L. infantum* WT (WT). Whereas, sera from some unvaccinated dogs infected naturally with *L. infantum* WT, recognized 21 kDa of *L. infantum* WT, but not of *L. infantum* H-line. Stationary phase of promastigotes (1×10^7^ cells per lane) were dissolved in sample buffer and run on a 12% SDS-PAGE gel. Blot was incubated with goat anti-dog IgG antibody HRP conjugated and then incubated with ECL Plus chemiluminescent substrate.

Two sera from vaccinated dogs which were seropositive at >1∶100, dogs V20 and V33, recognized the 21 kDa antigen of *L. infantum* H-line but not of *L. infantum* WT (Fig. 3). Ten out of 31 (32.2%) unvaccinated dogs which were seropositive at >1∶100 recognized the 21 kDa antigen of *L. infantum* WT, but not of *L. infantum* H-line (Table 1). As shown in Table 1, sera from 2 unvaccinated dogs, C2 and C61 were seropositive at >1∶100 but did not recognize the 21 kDa antigen of both *L. infantum* H-line and *L. infantum* WT. Sera from all dogs in both groups which were seropositive at ≤1∶100 did not recognize any antigens of *L. infantum* H-line or wild-type parasites.

### Clinical signs and leishmanial DNA analysis

The presence of leishmanial DNA and clinical signs of disease in vaccinated and unvaccinated dogs after 4 sandfly seasons are summarized in Table 1. No leishmanial DNA was found in the vaccinated dogs. In contrast, 9 out of 31 (29%) unvaccinated dogs were positive for the presence of leishmanial DNA over the 24 month period follow-up. As shown in Table 1, eight of 12 (66.7%) unvaccinated dogs with high levels of antibody (>1∶100) became PCR positive. The number of unvaccinated dogs that were PCR positive was significantly higher than that in the vaccinated dogs (*P*<0.002). All but 1 vaccinated dogs, [(2.2%), remained free of clinical abnormalities over the 24 months period of observation. Among the unvaccinated dogs, 9 out of 31 [(29%), two dogs, C8 and C1 in stages II and III of clinical signs of disease, respectively], presented one or more clinical signs of disease (Table 1).

### Report of VL cases from vaccine trial area

No VL cases were referred to the Afzalipour Medical Centre or recorded in the Centre for Disease Control and Prevention from the area of study more than 3 years since June 2010.

## Discussion

This is the first study to demonstrate efficacy of an attenuated *L. infantum* vaccine against natural CVL in dogs in a highly endemic area. The progression of leishmaniosis in dogs is associated with humoral response and depression of cellular immunity [27]. We previously reported that *L. infantum* H-line induced a CD4^+^Th1 response which was characterized by the production of relatively higher levels of IFN-γ and lower levels of IL-10 compared with those in the dogs infected with wild-type parasite [18], [19]. In contrast to *L. infantum* WT, the attenuated parasite was unable to multiply and survive in the visceral organs of immunized dogs [17] and remained localized in the skin at the site where the promastigotes were injected. It has been reported that promastigotes of *L. infantum* WT develop to amastigote forms in infected macrophages at the site of inoculation and the infection may spread, resulting in a systemic form [1]. Dissemination of the parasite in the visceral organs of *symptomatic* dogs is the result of the development of a non-protective Th2 response [28]. Subcutaneous vaccination with the attenuated line in the foreleg of the dogs, an area covered with hair, will prevent or significantly reduce the likelihood of uptake of attenuated line parasites by sandflies, which tend to feed only on areas of exposed skin. This observation alleviates concerns about the possibility of reversion to virulence by the attenuated line during passage through sandflies.

We reported subclasses of IgG in vaccinated dogs and correlated higher IgG2 with protection provided by *L. infantum* H-line [19]. In the present study, we found the level of IgG1 was significant lower than the level of IgG2 in the sera of vaccinated dogs. It has been reported that specific anti *Lieshmania* IgG1 in dogs is associated with the development of disease, whereas IgG2 antibody is associated with asymptomatic infection [29].

The present study was carried out in 3 villages, in the district of Dehsard, Baft County in the southeast of Iran highly endemic for CVL [3], [30], [31]. In a preliminary study, we found that 40.2% of the household dogs were seropositive for *L. infantum* (>1∶100). However, the prevalence of CVL in domestic dogs in this area might be higher than 40.2%. It has been reported that 37% of seronegative *asymptomatic* dogs from an endemic area were positive by the PCR with skin tissue [32]. It is recognized that introducing 103 dogs into an area could disturb the ecological dynamics between dogs, parasite and vectors. Householders whose dogs were seronegative did not allow their dogs to be used in the study. Thus dogs from a non-endemic area were brought in for the trial and whenever possible each household included unvaccinated and vaccinate dogs in order to equalize their degree of exposure to the risk of natural infection. The seroposivity of unvaccinated dogs was higher than that of household dogs, living in the area. It has been reported that some dog breeds such as the German shepherd are more susceptible to development of CVL [33], [34].

In the unvaccinated group 12 out of 31 dogs (38.7%) dogs were highly seropositive (>1∶100) which 35.5% of them developed signs of CVL over 24 months of period of monitoring. The specific anti-*Leishmania* IgG antibody was raised in the sera of the dogs vaccinated with *L. infantum* H-line [17], [19]. We found that except 2 unvaccinated dogs, all sera from the vaccinated dogs and unvaccinated dogs which were seropositive at >1∶100 recognized the 21 kDa antigens of *L. infantum* H-line or WT. It is in agreement with another study that band of 21 kDa has the highest immuno-reactivity and the most often recognized in the case of CVL [35]. In the present study we found sera from vaccinated dogs recognized the 21 kDa antigen of *L. infantum* H-line whereas, sera from unvaccinated dogs, which were natural infected with *L. infantum* WT recognized the 21 kDa antigens of *L. infantum* WT (Table 1). This observation is in agreement with our previous study that Western blot analysis of antibodies to the 21 kDa antigens of *L. infantum* H-line and WT is very useful method for distinguishing between dogs vaccinated with *L. infantum* H-line and dogs experimentally infected with *L. infantum* WT [20]. We found that sera from 2 unvaccinated dogs, C2 and C61, which were seropositive at >1∶100 did not recognize any antigens of *L. infantum* H-line or *L. infantum* WT (Table 1). It has been reported that the sensitivity and specificity of Western blotting is greater than IFA for diagnosis of CVL in dogs [25]. IFA cross-reaction antibody between *L. infantum* and other diseases such as *Ehrlichias canis* (*E. canis*) and *Babesia canis* and also some kind of clinical signs of disease might be possible [36], [37], [38]. Moreover, *E. canis* infection might induce immunosuppression [39] and therefore the immune system is not able to develop the protective immunity induced by the attenuated line and the vaccinated animal is not immune to natural challenge. This observation may be useful to explain the sign of disease in 2 vaccinated dogs,V20 and V33, over 24 months period monitoring and suggests in the future, we need to check for the presence of *E. canis* during vaccination with the attenuated *L. infantum*.

A number of vaccines such as FLM, LiESAp-MDP have shown a degree of effectiveness against experimental CVL in dogs [40], [41]. Our study is the first vaccine trial in dogs that might show an impact of a vaccine and in reducing the occurrence of VL in the local human population. It has been reported that human seropositive (>1∶800) in this area was 1.55% and approximately half of 108 registered cases were from Baft [3]. No VL cases were recorded from the area of study 3 years since these data were collected. Impact of this vaccine on human population should be confirmed in the further studies. The results presented clearly demonstrated that a gentamicin-attenuated line *L. infantum* vaccine induced a significant and strong protective effect against CVL in dog and holds considerable promise for vaccination of dogs against CVL in the field. 
