# Correction: Gentamicin-Attenuated *Leishmania infantum* Vaccine: Protection of Dogs against Canine Visceral Leishmaniosis in Endemic Area of Southeast of Iran

**DOI:** 10.1371/journal.pntd.0003787

**Published:** 2015-05-11

**Authors:** Hamid Daneshvar, Mohammad Javad Namazi, Hossein Kamiabi, Richard Burchmore, Sarah Cleaveland, Stephen Phillips

There is an error in affiliation 2 for author Mohammad Javad Namazi. Affiliation 2 should be: Sabzevar University of Medical Sciences, Sabzevar, Iran.
